# Next of Kin’s experiences of opportunities and limitations in user involvement: a qualitative study in a rural norwegian municipality

**DOI:** 10.1080/17482631.2026.2641798

**Published:** 2026-03-10

**Authors:** Erna Henriette Dahl Tyskø

**Affiliations:** a Department of Welfare and Participation, Western University of Applied Sciences, Bergen, Norway

**Keywords:** Next of kin, user involvement, municipal health and care services, family systems theory, rural municipality

## Abstract

**Purpose:**

User involvement is a fundamental value in Norwegian health and welfare policies. However, next of kin’s experiences of user involvement in municipal health and care services have not been sufficiently investigated in earlier research, despite the central role of the next of kin. The objective of this study is to explore how next of kin experience the opportunities and limitations of user involvement in municipal health and care services in a rural municipality in Norway.

**Materials and methods:**

A qualitative study was conducted using an interpretative phenomenological analysis of interviews with 20 next-of-kin participants (nine women and eleven men aged 38 to 90).

**Results:**

The analysis revealed four main findings: (1) between system resistance and opportunities- the struggle for recognition among next of kin, (2) between silence and support- information as an obstacle and opportunity, (3) between paperwork and collaboration- the limitations and potential of systems, and (4) between pressure and potential- the sense of responsibility in the next of kin role.

**Conclusions:**

The study demonstrates how relational dimensions and access to information are central to meaningful user involvement. The discrepancy between policy ambitions and everyday practice calls for strengthened, relationship-based facilitation strategies within municipal health and care services.

## Introduction

User involvement is widely recognised as a fundamental principle in contemporary health and welfare policy, both internationally (WHO, 2015) and in Norway (Norwegian Directorate of Health, 2017). In municipal health and care services, next of kin are involved both as “support persons” and as individuals with important information about the person to whom they are next of kin.

In this article, the term “next of kin” is used in accordance with the *Act on Patient and User Rights 1999*, Sections 1–3, and applies to the person whom the user has named as their next of kin or, if the user is unable to name a next of kin, the person who is next in a prioritised line of close contacts (such as friends or other relatives). The users referred to in this study receive services in accordance with the *Act on Patient and User Rights 1999,* Section 2-1(d) (user-controlled personal assistance), Section 2-1(e) (institutional stays), and Section 2−8 (particularly burdensome care tasks), as well as services stated in Sections 3−1 through 3−6 of *The Act on Municipal Health and Care Services etc. 2011*. These services include home nursing care, respite care, follow-up services, and institutional stays. The next of kin of receiving municipal health and care services often play a crucial role in the services received. This type of informal care is prevalent and is estimated to occur on the same scale as the professional care provided by municipal services in Norway (Ministries, 2020). As an informal care provider with in-depth knowledge of the user’s needs and wishes, the next of kin is an equal and essential partner for municipal health and care services.

*“User involvement implies that those who are impacted by a decision, or who are using services, have a say in decision-making processes and the design of the services provided” (Ministry of Health and Care Services, 1996–1997,* p. *29). Although the term service “user” may be perceived as impersonal, it is utilised here in accordance with current legislation.*

Previous research has identified several barriers to realising policy ambitions of user involvement in practice. Practical obstacles such as time constraints, unclear divisions of responsibility, and limited opportunities for participation may hinder the effective involvement of next of kin in decision-making processes (Chen & Beresford, 2023; Hjelle et al., 2016). Studies further indicate that involvement in health and care services often takes place on professionals’ terms and may be experienced as fragmented and limited (Carlsen & Lundberg, 2017; Husebø et al., 2017; Nakrem & Hynne, 2017; Larsen et al., 2018). More recent research highlights how institutional structures and documentation requirements can shape and sometimes constrain user participation, whereas dialogical practices may create greater opportunities for meaningful involvement (Solberg et al., 2025). At the same time, next of kin are frequently described as important resources with valuable knowledge of users’ everyday lives. Nevertheless, their role in decision-making processes often remains unclear, reflecting a persistent gap between policy ambitions and everyday practice. To further explore these tensions between policy ideals and lived practice, theoretical perspectives on participation provide a useful analytical framework. In response to these tensions between policy ambitions and everyday practice, collaboration is frequently highlighted as a central principle in the development of healthcare services.

Collaboration is highlighted as an overarching principle in the development of healthcare services (Andreassen, 2005; Kjellevold, 2006), which signals a clear expectation of the active involvement of the next of kin. In line with this, the next of kin appears to be a natural and important element of what is often referred to as an extended user perspective. Arnstein’s (1969) “ladder of participation” provides a foundational framework for understanding degrees of user involvement, ranging from symbolic participation to genuine influence and shared power. The model underscores that participation entails more than consultation; it requires the opportunity to affect decisions. Applied to the involvement of next of kin, Arnstein’s framework highlights the potential gap between formal inclusion and actual influence in practice. Tritter (2009) challenges the linear assumption of increasing influence and instead conceptualises participation as a dynamic and contextual process across three dimensions: individual versus collective, direct versus indirect, and proactive versus reactive involvement. In municipal health and care services, next-of-kin participation often occurs in informal and indirect forms, mediated through professionals rather than through direct influence in decision-making arenas. Together, these perspectives illuminate tensions between ideals of collaboration and the often-fragmented realities of user involvement.

Despite the central role of next of kin in municipal health and care services, their experiences of user involvement remain insufficiently documented. Existing research is limited and often based on specific service contexts, such as nursing homes (Larsen et al., 2018; Nakrem & Hynne, 2017), indicating restricted insight into involvement across diverse municipal services. Moreover, although the legal position of next of kin has been strengthened, reports from next of kin and their organisations suggest continuing challenges in safeguarding their rights and needs (Ministries, 2020). There is therefore a need for in-depth exploration of how next of kin perceive both opportunities and limitations in user involvement. This study seeks to address this gap by examining next-of-kin experiences within municipal health and care services. The study’s research question is: *how have next of kin experienced the opportunities and limitations of user involvement in municipal health and care services in a Norwegian regional municipality?*

## Method

The study was conducted in a rural municipality of around 10,000 inhabitants, characterised by a large geographical catchment area and considerable travel distances. These structural conditions influence the organisation of municipal health and care services and shape opportunities for next-of-kin involvement. A qualitative approach was used to gain in-depth insight into the next of kin’s experiences of user involvement in municipal health and care services. Interpretative phenomenological analysis, rooted in phenomenology and hermeneutics, was chosen for this analysis. Such a design is well-suited for shedding light on the complex interplay between individual experiences and contextual frameworks. It also allows for the discovery of nuances, variations, and depth in how the next of kin experience user involvement in these services (Brinkmann, 2024).

### Inclusion criteria and selection

The participants had to meet the following criteria: (1) be next of kin to a person receiving municipal health and care services and (2) be over 18 years of age and competent to provide consent. There were no requirements regarding duration of next-of-kin involvement. Access to an overview of relevant next of kin was approved by the head of municipal health and family services. A total of 579 invitations were distributed, of which 37 individuals responded. A strategic selection was made to ensure representation across relevant service areas, including nursing homes, respite care for children, home care nursing, and follow-up services. Twenty participants were included in the study; the remaining respondents did not meet inclusion criteria, withdrew, or were unavailable for interview.

The final sample consisted of 20 next of kin. Participant characteristics are summarised in Table I. Participants had varied relationships to the service users, including parent, partner, adult child, sibling, and other close relations. To ensure variation in care contexts, the sample included next of kin to children receiving respite services, adults with long-term care needs, and older persons.

**Table I.** Characteristics of participants (*n*=20).

Gender	Age range (years)	Residence in relation to user	Employment situation
Women (*n*=9)	38–91	Same municipality (*n*=16)	Employed full time (*n*=10)
Men (*n*=11)		Different municipality (*n*=4)	Pensioners (*n*=8)
			Welfare benefits (*n*=2)

Despite the relatively low response rate, the sample was considered diverse in terms of gender, age, and socioeconomic background. Participants received written information and provided informed consent prior to the interviews.

Detailed individual characteristics are not disclosed due to confidentiality considerations in a small municipal context. Participants are identified by numbers in the quotations presented in the results section. The numbering reflects the idiographic orientation of IPA, emphasising attention to each participant’s distinct lived experience.

### Qualitative interviews

Semi-structured interviews were conducted to collect data. The interview guide was developed collaboratively by the author and principal supervisor. The purpose of the guide was to shed light on the experiences of user involvement among the next of kin. The interviews addressed four main themes: (1) experiences where next-of-kin knowledge is taken into consideration, (2) experiences of the services provided, (3) experiences of involvement, and (4) experiences of professional judgement. The interviews were conducted over 7 weeks, and the duration of the individual interviews ranged from 65 to 85 minutes.

### Researcher position and preunderstanding

The author was employed by the organisation in which the study was conducted and was also raised in the regional municipality where the study took place. As a result, some participants were known to the author prior to the interviews. This positionality required careful reflection on potential strengths and limitations related to familiarity and insider knowledge. To minimise potential bias, the selection process was conducted in collaboration with the principal supervisor, who also conducted five interviews in cases where prior relationships could pose a challenge.

### Interpretative phenomenological analysis (IPA)

The material underwent interpretative phenomenological analysis (IPA), the purpose of which is to gain an understanding of how people create meaning through their own experiences (Lorås, 2024; Smith et al., 2022). IPA has three central pillars: phenomenology, hermeneutics, and ideography. Phenomenology emphasises participants’ subjective experiences without imposing premature interpretations (Binder et al., 2024). IPA also recognises that the researcher’s understanding will always be characterised by interpretation and that meaning is created through these interpretations (Lorås, 2024). The hermeneutic aspect deals with interpreting and translating experiences, whereas the ideographic approach emphasises the individual’s unique lifeworld and context (Binder et al., 2024).

IPA requires continuous reflection on one’s preconceptions to avoid preconceptions that interfere with the analysis. The analysis is characterised by a reflective practice in which the shift between interpretations and the empirical material helps maintain proximity to the participants’ lifeworld while also allowing for a deeper understanding of their experiences. This study uses an inductive approach in which the analysis process is driven by the data (Lorås, 2024).

### The process of analysis in six steps

The analysis followed six steps, inspired by Dallos and Vetere (2005) and Lorås (2024):

1. Review and in-depth study: The transcripts were reviewed multiple times to allow sufficient familiarisation with the material and ensure that the participants’ voices remained the key point. Impressions and reflections were noted in this way. One common theme that stood out at an early stage was how participants expressed uncertainty about what services existed and how they functioned.
2. Exploratory comments: The next step involved free-text comments on the choice of words, content, and manner of speech. The aim was to allow for different interpretations without making premature assumptions.
3. Experience-based and interpretative comments: Initial comments were further processed and elevated to a more analytical level. The focus shifted from the transcription itself to the comments, and the meaning was interpreted in light of the context and its possible underlying significance.
4. Coding: The key codes were numbered and documented with reference to the interview, page, and line number to ensure traceability and overview.
5. Categorisation: The codes were physically laid out and reviewed to identify patterns and correlations. Codes with common meanings were collected and formed the basis of the thematic units.
6. Naming the main findings: The analysis resulted in four main findings that can be summarised as: (1) between system resistance and opportunities—the struggle for recognition among next of kin; (2) between silence and support—when information hinders and invites user involvement; (3) between paperwork and collaboration—when systems limit and enable user involvement; and (4) between pressure and potential for user involvement—stress and sense of responsibility among next of kin.

### Research ethics

The study was approved by the Norwegian Agency for Shared Services in Education and Research (SIKT) (reference no. 163929). All participants signed informed consent forms and were informed that they could withdraw at any time without having to state a reason. Audio recordings and anonymised transcripts were stored on a secure server at Western Norway University of Applied Sciences (HVL). No sensitive health information was collected; however, as the study was conducted in a small municipality, certain descriptions may still entail a risk of indirect identification. All participants were verbally informed of this risk prior to the interviews. The benefits of conducting this study exceeded the potential risks of identification.

### Limitations

Although 579 requests for participation were sent to next of kin, only 37 responses were received. There is reason to assume that those who chose to participate had more powerful experiences than others in terms of either satisfaction or dissatisfaction with the services they were in contact with. This may have contributed to a certain bias in the sample and raises questions about whether the findings are representative of the broader group of next of kin in rural municipalities in Norway.

Municipal health and care services are broad, ranging from home-based services to respite care for children. It is reasonable to assume that such differences may influence the experiences of user involvement among the next of kin. Although this was a methodological limitation, it was assessed that such variations were not critical for the purpose of the study. The aim was to shed light on how user involvement was experienced by the next of kin across types of services rather than to compare experiences based on the nature or scope of the services.

## Results

This study explores what the next of kin to persons receiving municipal health and care services in a Norwegian regional municipality perceive as limitations and opportunities for user involvement. On the basis of interpretative phenomenological analysis (IPA), four findings were identified that can be summarised as follows: (1) between system resistance and opportunities- the struggle for recognition among next of kin; (2) between silence and support- when information hinders and invites user involvement; (3) between paperwork and collaboration- when systems limit and enable user involvement; and (4) between pressure and potential for user involvement- stress and sense of responsibility among next of kin.

### Between system resistance and opportunities- the struggle for recognition among next of kin

This finding indicates that the next of kin felt that they had to fight to be heard and understood in their encounters with municipal health and care services. Many described this as an ongoing struggle that took place in meetings, when writing applications, in complaint processes, during telephone conversations, and in informal contact with these services. This struggle was described as background noise in their day-to-day lives. As Participant 12 stated, “It does something to you. You have nights where you lie awake thinking, is this how the future is going to be? That you constantly have to fight and search.” The struggle was not only about being granted specific services but also about being treated with dignity and recognition. At the same time, several next of kin described an unrealised potential for user involvement. They indicated that their interactions with municipal health and care services would have been easier and more targeted if they had been seen and heard when collaborating on services for the person to whom they were next of kin.

Several next of kin described experiences of good collaboration. Participant 14 stated, “There was one time they actually came to my home and said, ‘We need your help to understand what works.’ That was the first time I felt that my voice had mattered.” Such experiences show that it is possible to establish dialogue when the next of kin’s knowledge of users’ services is taken into consideration. At the same time, some described being in a skewed power dynamic state. Even in cases where the user’s care challenges were known, their needs had to be documented again, and the next of kin’s knowledge as a caregiver was rarely validated. Participant 1 said, “The most stressful thing is all the paperwork. […] You sit there and write complaints and complete applications, or you have to document all things. It’s awful.” These experiences illustrate how genuine user involvement requires that next-of-kin experiences be professionally valued and have a genuine influence on decision-making processes.

Several next of kin described their emotional and physical consequences. Participant 4 stated, “We have spent a lot of time and energy arguing with the municipality. I am struggling with my mental health issues. […] It really gets to you when you are in a situation like this.” Nevertheless, many next of kin expressed a clear desire and willingness to contribute. To feel that they were being heard, they developed their own strategies in their encounters with municipal health and care services, such as approaching service providers in a pragmatic manner, with knowledge of legislation and policy guidelines relating to healthcare services. However, the next of kin were also worried about being perceived as demanding. They were happy to contribute and went to great lengths for the person to whom they were next of kin, preferably in collaboration with health and care services, if this was facilitated. For a certain next of kin, this responsibility strengthened their bond with the user, and fighting on behalf of the user became an important part of the next of kin’s life. However, others felt that they were left on their own when the user did not want to participate in dialogue with the services or was incapable of doing so. This created challenges, particularly when the assessments of the next of kin contrasted with the user’s wishes or lack of insight. Participant10 stated:

*Well, I think that ... in this type of situation, with this type of patient, the smartest thing to do might be to listen more to family members than to the patient. When a patient does not know what is best for them. […] So, I think you have to consider the fact that it might actually be wise to sometimes listen a little more to family members than to the patient.*

Others shared similar experiences, where communicating the need for help became especially difficult because the users themselves did not want help or refused to acknowledge their own challenges. As Participant 8 stated, “I had to deal with this on my own because he did not want help. So, you sit in these meetings and feel like you are failing on both counts. Either you are failing him, or you are not telling the truth.” This type of experience also illustrates how the next of kin must balance consideration for the user’s autonomy with their need for essential assistance. This means that the ability of the next of kin to contribute may be limited if the user does not want their involvement, which makes it difficult to balance participation in practice. In cases where the user did not wish to or was unable to express themselves, the next of kin felt that the services should have done more to ensure that their voice was heard in the decision-making processes without compromising the user’s autonomy. Structures are required to capture relational nuances and allow room for discussion and reflection. Thus, the experiences of next of kin highlight the need to avoid viewing user involvement as an unambiguous individual right. When service providers are able to listen to and validate the experiences of the next of kin, even when they deviate from the user’s perspective, this enables a more realistic and relationally rooted practice of user involvement and collaboration.

### Between silence and support- when information hinders and invites user involvement

This finding concerns what next of kin described as receiving too little information about their rights and the services that they could expect and request for the person to whom they were next of kin. Many next of kin had experienced navigating a complex and confusing system on their own. Participant 20 stated, “It took many years before we realised that it was possible to apply for respite care […]. I did not think it applied to me at the time.” The information that the next of kin receives is often perceived as random, fragmented, and difficult to access. Some learned about opportunities through chance encounters with others in the same situation, while others found that service providers’ communication was characterised by sector jargon. Participant 4 described this as follows: “We’ve actually felt neglected by the municipality the whole way. However, we have regular meetings. For us, the flow of information has been very poor.” Many said that they spent a great deal of time and effort searching for information on their own. Online searches, phone calls, emails, social media, and conversations with others in similar situations are part of their daily lives. Some became, as they described it, “experts on the system”, not because of but in spite of the assistance they received. Others indicated that a lack of an overview led to resignation. As Participant 15 stated, “A checklist would have been great, I think […], at least when you first come into contact with home nursing care and are in that position.” Several next of kin would have liked to have a contact person who could provide information that was adapted to their situation and communicated in the language they understood and in a way that acknowledged their role. They felt that if information was provided in the right way, with respect and accessibility, it would have strengthened their sense of security and scope for action. In cases where the next of kin felt that they had received tailored information, it provided them with a space to participate in the design and assessment of the services as collaborative partners. Many next of kin expressed wanting to share responsibility rather than lose it altogether.

The experiences of next of kin also indicate that access to information was unevenly distributed. Those with a higher education, knowledge of laws and regulations, good language skills, or experience with healthcare services were often in a better position to understand and utilise the services. Participant 18 stated:

*I searched for it myself. […] I received a rejection, but then I went through the regulations point by point. […] They thought someone had helped me because my replies were thorough. But I’d do it all myself. I thought that people who are not able to do this themselves have lost.*

Being the next of kin while simultaneously navigating a confusing system was experienced as a double burden by many people. In addition, many felt an underlying anxiety about whether they had done enough, understood enough, or perhaps missed out on important assistance simply because no one had informed them of the services they might be entitled to. Many of the next of kin said that it was not just about getting the right information; it was also about being seen and included. When someone took the time to explain or ask what they needed, it made a significant difference. As Participant 6 stated, “She explained it in a way I could understand, and it was the first time I actually realised that we could apply for more than what we had.” These experiences indicate that information is not solely about rights and procedures but also about relationships and roles. When information is provided in a way that recognises the experiences and position of the next of kin, it strengthens their sense of security and ability to contribute. Thus, the findings point to an important area for development: user involvement requires not only open doors but also an invitation, structure, and language that enables the person to enter and participate.

### Between paperwork and collaboration- when systems limit and enable user involvement

Many next of kin felt that municipal health and care services were largely unresponsive to individual needs. The services were portrayed as being focused on procedures and financial frameworks. In their meetings with the municipality, the next of kin described conversations largely characterised by forms, standardised assessments, and limitations rather than dialogue, flexibility, and recognition. Participant 12 stated:

*It is quite painful to observe this. For example, when filling out these forms, you must translate everything into the language they understand. There is no room to add things that are difficult to put into the words. And I feel that when we do that, we are losing something. We’re losing her. She becomes an application and not a person.*

Several of the next of kin said that such experiences not only hindered user involvement but could also be perceived as degrading and disempowering. Bureaucracy is evident in many daily situations. The next of kin had to complete extensive forms and participate in surveys that were perceived as largely irrelevant and difficult to understand. Tools intended to ensure equal treatment became simplifications that did not capture the complexity of life. Participant 15 stated, “There are so many standards and boxes to tick, and such, but there’s no room for what actually happens in our homes.” A specific burden arises when the next of kin has to complete certain forms on the user’s behalf. Several described this as a dehumanising process where intimate and personal details had to be translated into brief and neutral formulations. Those in need of assistance were reduced to a case rather than being recognised as people with different needs. Their relational and vulnerable aspects did not fit into boxes and figures. Some participants described the discomfort and shame of having to make a user’s needs measurable and adapt them to the language of the system. Filling out forms became not only a practical task but also an emotional and ethical burden.

While such forms were intended to create structure and fairness, in practice they obscured the uniqueness of each situation. Several of the next of kin stated that their own experiences were overlooked, and their voices were valued less than what was written in the forms. Encounters with municipal health and care services often left them feeling that they were speaking a foreign language. The next-of-kin participants tried to convey their day-to-day problems, while service providers responded with paragraphs, forms, and financial frameworks. Participant 3 described this as follows:

*In the end, you tell them what they want to hear. You learn that. You know what to write about in the application to get a yes. It is no longer about what we need but about what the system accepts. And that hurts. This implies that we are no longer honest. However, it feels that we have no choice.*

The next of kin described a strong need for service providers who listen to openness and respect and recognise the next of kin’s insights as valuable knowledge. Next-of-kin participants called for room for dialogue, individual adaptation, and flexible assessment based on the entire family’s situation.

Certain next of kin also reflected on and indicated that they understood why municipal health and care services need structure and documentation. Rigidity was therefore understood not only as a choice by service providers but also as an expression of prioritisation, efficiency requirements, and transfer of responsibilities. As Participant 16 stated, “Eventually, you give up. There is no point in trying it any more. You just take what you can get.” These realisations led to an emotional paradox: understanding on the one hand, grief and powerlessness on the other. For many, what was intended to ensure quality was a barrier to flexible and relational assistance. When the personal and the relational were hidden behind paperwork, this weakened not only the next-of-kin role but also trust in municipal health and care services. These experiences highlight an important development area. When services allow for dialogue, adaptation, and recognition of the perspectives of next of kin, user involvement can be realised in practice. However, service providers in municipal healthcare services must take the first step of listening, inviting, and acknowledging.

### Between pressure and potential for user involvement- burden and sense of responsibility in the next of kin role

This finding relates to how being the next of kin who is in contact with the municipal services is not solely about providing care for the service recipient, but also about bearing an extensive and sometimes burdensome responsibility, both for the person they are close to and for ensuring that the system actually works. For many, this has led to a life of constant vigilance, including follow-ups, reminders, cheques, complaints, and prevention. Participant 6 stated:

*I often feel that I am one who has to keep up with things. I am the one who has to check things, and I am the one who has to make phone calls. If I do not do it, nothing will happen. […] But, I do it because I want him to be okay. Not because I really want to be a coordinator.*

This sense of responsibility is often obscured and unrecognised. Several next-of-kin participants felt that the burden might have been reduced had they been perceived as a resource to a greater extent. As Participant 9 stated, “I’m the one who knows him best. I know what he needs and how things fit. If they had asked more questions, we might have been given better solutions.” When personnel listened, acknowledged the efforts of the next of kin, and involved them in assessments and decisions, responsibility became easier to bear. The experience of next of kin indicates that user involvement does not necessarily imply that service providers must do more. It is about sharing responsibility and being valued as a partner.

The role of the next of kin often develops gradually. The list of tasks—from day-to-day logistics and medical follow-up to contacting professionals and working on applications—has grown ever longer. Several studies indicate that it is crucial for service providers to take time to listen to and acknowledge their contribution. Being seen as a partner and not just as a caregiver makes the responsibility less lonely. Participant 11 stated:

*She took time to ask me what it was like to be next of kin. Not just what I knew about my son but how I felt. This was the first time that anyone had ever done so. And that made me feel a bit more like a person and not just like a resource.*

When the next of kin were recognised as collaborative partners who had insight and responsibility, their workload was not necessarily reduced, but it became easier to bear because the responsibility was shared.

Several next-of-kin participants told us that being constantly on call affected their own health, working lives, and social relationships. When mistakes or failures occurred, it was often the next of kin, and not the service providers, who had to fix things. Simultaneously, the next of kin talked about service providers who accepted more responsibility and followed up in ways that inspired trust and provided relief. Participant 3 said, “She, the caseworker, took the time to listen to what we had experienced. This resulted in all differences. We did not feel like just another case in the pile.” These experiences show that collaboration is possible, and that user involvement can work when relationships, experience, and responsibility are taken seriously.

Several next of kin said that, over time, they developed considerable expertise and confidence in their roles. They learned to navigate the services, understand the regulations, make a case for their needs, and express themselves in the language of the services. This expertise was developed through experience and indicated an untapped potential, as many of the next of kin had insights that could strengthen the services if they were invited in. As Participant 9 stated, “We know the user best. If they were only asked, we could have saved them both time and mistakes.”

Although many described the feeling of being alone, several also spoke of encounters with service providers who listened to them and facilitated collaboration. Often, it was not the larger measures but rather the smaller actions that gave them a sense of being part of a community—being remembered, getting a phone call, or being asked for advice. Participant 8 said, “She just said that we cannot do this without you, but we’d like to hear how you are doing too. This was the first time I felt that we were actually on the same team. And that made it easier to cope.” Such experiences indicate that user involvement is not necessarily about access to more services but about the importance of a good working relationship. When the system recognises the efforts of the next of kin and seeks their perspectives, their relationship transforms from struggle and control to mutual support and shared responsibility. It is, therefore, a matter of not only reducing responsibility but also sharing responsibility with someone else.

## Discussion

This study clarifies that user involvement in municipal healthcare services in a Norwegian regional municipality is a complex and relational phenomenon. Although the right to user involvement is clearly anchored in both legislation and national policy guidelines (Act on Patient & User Rights, 1999; Ministry of Health & Care Services, 1996–1997; health & Care Services Act, 2011), there is considerable uncertainty about how involvement is actually practised in municipal health and care services. This is particularly evident in the next of kin’s descriptions of their encounters with the municipal health and care services of a system characterised by varying degrees of influence and recognition.

The next-of-kin participants described struggling to be heard and understood in their encounters with the service providers. Several said that they struggled to access information about available services, that the user’s individual needs were not adequately met, and that they felt a strong and persistent sense of responsibility toward the user. These findings can be illustrated by Arnstein’s (1969) normative ladder of participation, with user involvement understood in terms of degrees of power and influence, from non-participation to genuine co-determination. Several next of kin felt that their participation was on the lower rungs of the ladder, indicating symbolic participation without genuine influence on decision-making processes. This perspective can be further nuanced by Tritter and McCallum (2006), who criticise Arnstein’s model as being too static and hierarchical. They introduced a more dynamic approach in which user involvement is perceived not as a linear climb up a ladder but rather as movements in different directions, as in a board game with players moving from side to side, crossing roads, and climbing ladders. Based on this model, the experiences of next of kin appear unpredictable and fluctuate between different forms of influence, with the degree of genuine user involvement depending on structural frameworks, relational conditions, and the ability of service providers to collaborate. In this study, the next-of-kin participants described experiences ranging from symbolic user involvement, where participation gave the impression of influence without genuine effect (Arnstein, 1969), to genuine user involvement, where they were actually heard and involved in service decisions. When participation appears to be a formality rather than genuine collaboration, the next of kin may feel that they are involved merely for the sake of it rather than for actual influence. Some next of kin found that speaking up on behalf of the user could be perceived by the services as disloyalty. This illustrates how relational dynamics are closely tied to structural frameworks, including the legal rights anchored in Section 3−1 of the Patient and User Rights Act. This creates a gap between the expectation of genuine influence and the actual power to influence, which is frustrating. Tritter and McCallum (2006) warn against instrumental approaches, where user involvement is used solely as a method for achieving organisational goals, without any real transfer of power. They emphasise that dialogue and contextually adapted collaboration are needed to ensure meaningful participation. This is particularly relevant in municipal health and care services, where the next of kin describe standardised procedures and resource constraints. When next of kin feel that their participation is reduced to filling out forms or providing input that is not followed up, their involvement falls within the instrumental level. This is stressful because it creates a gap between the expectation of influence and the actual power to have influence. When service providers ask about and show consideration for the experiences of next of kin, this can be interpreted as genuine user involvement, in which a transactive form of user involvement promotes shared responsibility and trust. This not only strengthens the relationship between next of kin and service providers but also improves the quality of municipal health and care services.

Access to information is crucial for user involvement. Several next-of-kin participants indicated that it was challenging to navigate the confusing system. The confusion was due to communication characterised by sector jargon, with little information available about municipal health and care services and what they entail. This created frustration and made it difficult to ascertain the services and measures to which the person to whom they were next of kin might be entitled. However, when information was provided early and clearly and adapted to the specific situation, the next-of-kin participants felt more secure about both the services provided and the measures they could request. This is supported by previous studies that emphasise that accessible and relevant information is a prerequisite for genuine user involvement (Chen & Beresford, 2023; Hjelle et al., 2016).

These findings also highlight gradual changes in the role of next of kin. The role has changed from support person for the user to “spokesperson” and coordinator for the user’s needs. This change can be understood in light of family systems theory, which emphasises how multiple systems affect the health and quality of life of each individual family member (Priest, 2021). This theory also addresses how rules and patterns in the family system can make it difficult for some family members to cope with stress, such as when a loved one falls ill and is no longer able to make all the decisions for themselves. Such burdens can reduce a family’s ability to adequately navigate municipal health and care services. According to general systems theory (von Bertalanffy, 1968), this gradual change in the next-of-kin role may be understood as a response to shifts in municipal health and care services. When no decisions are made regarding services that the next of kin perceives as essential, they often step in to fill the gaps, which means that the genuine need for assistance may not be identified. Without clear dialogue regarding the contributions of the next of kin, there is a risk that the next of kin will assume extensive care tasks that are not acknowledged by the services. At the same time, this study has found examples of good collaborative processes, where next of kin said that they felt valued and that their voices were important in the collaboration for the user. This is in line with research emphasising the importance of a relational approach and flexible forms of collaboration (Chen & Beresford, 2023; Mulquiny & Oakman, 2022).

The rural context of this study is likely to have influenced the findings. Characteristics such as large geographical distances, limited service capacity, and closer interpersonal relationships between actors create both opportunities and constraints for next-of-kin involvement. On the one hand, proximity can foster trust and flexibility; on the other hand, limited resources and overlapping roles may reinforce structural barriers. Improving next-of-kin involvement may also come at a cost to patient involvement, depending on the power dynamics between the patient and their next of kin. This underscores the importance of balancing different actors’ voices and ensuring that strengthening next-of-kin involvement does not unintentionally weaken the patient’s role. This study confirms previous research showing a significant gap between ideal and actual practices in terms of user involvement (Husebø et al., 2017; Tyskø et al., 2024). This is particularly true in smaller municipalities with limited access to professional expertise (Ministries, 2020). When the next of kin is not guaranteed genuine influence over the services provided to the user, user involvement is reduced to an ideal policy unrooted in the practice of municipal health and care services. Genuine user involvement requires more than the provision of information. It requires opportunities to participate, be heard, and be met with respect.

## Concluding comments

This study presents next-of-kin experiences of user involvement in municipal health and care services that are characterised by both opportunities and limitations. Recognition, access to information, and the quality of the relationship with service providers were decisive in how involvement was experienced. Although policy guidelines emphasise user involvement, this often seems unpredictable and fragmented. The findings indicate a need for more flexible and relationship-oriented forms of collaboration, where both the insights and the role of the next of kin are valued and better integrated into the services.

### Implications for practice and research

To ensure genuine user participation for the next of kin, practices must be developed for user involvement that is characterised by collaboration, dialogue, flexibility, and recognition. For user participation to be more than an ideal, municipal health and care services need to acknowledge next of kin as partners. User involvement should be viewed as a continuous relational process rather than a formality. This requires service providers to develop skills in communication, relationship building, and flexible interaction—particularly in encounters with next of kin who are facing challenging life situations.

Services should strengthen their expertise in what it means to be next of kin and how next of kin’s contributions can be meaningfully integrated into decision-making processes. Such a shift has the potential to enhance both service quality and the trust between next of kin and service providers. Based on these findings, I suggest clearer national and local policies that define responsibilities and procedures for next-of-kin involvement. This includes establishing structured arenas for dialogue, clearer role descriptions, and formalised expectations for collaboration.

At the practice level, there is a need to improve communication routines, support healthcare leaders in navigating complex relational dynamics, and provide targeted training that enhances competence in user and next-of-kin involvement. However, strengthening next-of-kin involvement also requires careful attention to power dynamics and patient autonomy. Practices should aim to enhance collaboration with next of kin without undermining the user’s voice or decision-making authority.

Future research should examine how user involvement is practised in different municipal contexts and how the role of next of kin evolves over time. Further knowledge is also needed on the factors that hinder or promote collaboration between next of kin, users, and service providers.

## Data Availability

Data supporting this study consist of qualitative interview transcripts collected for health and well-being research. These data contain sensitive information and potentially identifiable details; therefore, the complete dataset is not publicly available, to ensure participant confidentiality and compliance with ethical and legal research standards. De-identified excerpts that are central to the analysis and findings are included in the article and its supplementary materials. Full access to the de-identified dataset may be granted upon reasonable request, subject to satisfying appropriate data protection regulations and ethical approvals. Interested researchers may contact the corresponding authors to initiate such requests.
